# Dissecting the Genetic Components of Adaptation of Escherichia coli to the Mouse Gut

**DOI:** 10.1371/journal.pgen.0040002

**Published:** 2008-01-11

**Authors:** Antoine Giraud, Safia Arous, Marianne De Paepe, Valérie Gaboriau-Routhiau, Jean-Christophe Bambou, Sabine Rakotobe, Ariel B Lindner, François Taddei, Nadine Cerf-Bensussan

**Affiliations:** 1 INSERM, U571, Paris, France; 2 Université Paris Descartes, Faculté de Médecine René Descartes, IFR94, Paris, France; 3 INRA UEPSD, Jouy-en-Josas, France; 4 Department of Medical Biochemistry and Microbiology, Uppsala University, Uppsala, Sweden; 5 INSERM, U793, Paris, France; University of Toronto, Canada

## Abstract

While pleiotropic adaptive mutations are thought to be central for evolution, little is known on the downstream molecular effects allowing adaptation to complex ecologically relevant environments. Here we show that Escherichia coli MG1655 adapts rapidly to the intestine of germ-free mice by single point mutations in EnvZ/OmpR two-component signal transduction system, which controls more than 100 genes. The selective advantage conferred by the mutations that modulate EnvZ/OmpR activities was the result of their independent and additive effects on flagellin expression and permeability. These results obtained in vivo thus suggest that global regulators may have evolved to coordinate activities that need to be fine-tuned simultaneously during adaptation to complex environments and that mutations in such regulators permit adjustment of the boundaries of physiological adaptation when switching between two very distinct environments.

## Author Summary

The mammalian intestine is a privileged physiological site to study how coevolution between hosts and the trillions of bacteria present in the microbiota has shaped the genome of each partner and promoted the development of mutualistic interactions. Herein we have used germ-free mice, a simplified albeit ecologically relevant system, to analyse intestinal adaptation of a model bacterial strain, Escherichia coli MG1655. Our results show that single point mutations in the *ompB* master regulator confer a striking selective adaptive advantage. OmpB comprises EnvZ, a transmembrane sensor with a dual kinase/phosphatase activity, and OmpR, a transcription factor controlling more than 100 target genes. In response to environmental changes, EnvZ modulates the phosphorylation and thereby the transcriptional activity of OmpR. We further show that the selective advantage conferred by OmpB mutations is related to their additive and independent effects on genes regulating permeability and flagellin expression, two major set of genes controlled by OmpR. These results suggest that global regulators may have evolved to coordinate physiological activities necessary for adaptation to complex environments and that mutations offer a complementary genetic mechanism to adjust the scale of the physiological regulation controlled by these regulators in distinct environments.

## Introduction

Bacterial populations are powerful model to explore the mechanisms of evolution. Several in vivo experiments have pointed to the possible important role of pleiotropic adaptive mutations, but their molecular basis remain in most of cases largely elusive [1–3]. Here we have used gnotobiotic mice that offer a simplified and controlled albeit ecologically relevant experimental environment model to analyse the adaptation of E. coli MG1655 to the gut, as E. coli is usually the first colonizer of the mammalian newborn germ-free intestine [4,5]. Taking advantage that this laboratory strain is entirely sequenced and easily accessible to genetic manipulations, we could design a study that allowed deciphering the beneficial effects of pleiotropic mutations during intestinal colonisation.

The mammalian intestine is a privileged physiological site to study how coevolution between hosts and the trillions of bacteria present in the microbiota has shaped the genome of each partner and promoted the development of mutualistic interactions. Genetic adaptation to the host over the millions years of coevolution has translated into physiological regulatory pathways that are rapidly mobilized in response to intestinal colonization [6–9]. In the microbiota, the contrast between the considerable number of species, more than a thousand, and the small number of bacterial divisions [10], indicates that coevolution has selected bacterial genera possessing the genetic gear to adapt to the host environment, a notion supported by recent evidence that gut habitats in different host species dictate distinctive structures of intestinal bacterial communities [11]. Yet, the intestine is a complex and highly dynamic ecosystem composed of a large diversity of niches that vary in space and time, where bacteria face a permanent adaptive challenge. Furthermore, intestinal bacteria must be able to hurdle between their hosts across the exterior environment and for certain such as E. coli to switch between two entirely distinct natural environments. Gnotobiotic animals that offer a simplified albeit relevant model to study reciprocal mechanisms of adaptation between bacteria and their hosts, within a few days, the host can only adapt via physiological changes, whereas bacteria can adapt both by gene regulations and adaptive mutations. Indeed, we have previously demonstrated that adaptive mutations are central for efficient intestinal colonization by *E. coli* MG1655 [12]. Here we show that adaptation of this strain of *E. coli* during intestinal colonization entails rapid and parallel evolution in the EnvZ/OmpR two-components transduction system [13]. The gain of fitness provided by the diverse mutations selected in this global regulator during in vivo colonization results mainly from two distinct and measurable effects on motility and permeability that are both reduced in the mutant strains selected in the gut environment. These findings suggest that evolutionary pressures can put a diverse set of physiological functions facilitating adaptation under the control of one global regulator, and that mutations permit to adjust the scale of the physiological regulation controlled by this regulator in a given environment.

## Results

### E. coli Strains Selected during In Vivo Colonisation Exhibit a Reduced Motility Phenotype That Results from Mutations in the EnvZ-OmpR Regulator System

**Figure 1:**
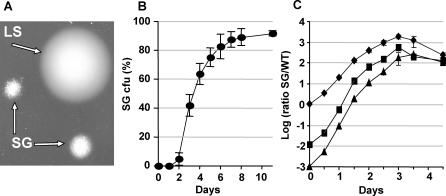
A New Colony Morphotype Is Rapidly Selected during Colonisation *(*A) Morphotypes observed on motility plates: large smooth (LS; similar to the ancestral MG1655 strain) and small granular (SG; selected mutant). (B) Evolution over time (in days) of SG colony forming units (mean (cfu) ± standard deviation) in the feces of 12 mice. (C) Evolution over time (in days) of the ratio of green to red cfu in the feces of mice inoculated with MG1655 ptet-GFP *ompBSG1-cat* (containing the SG1 *envZ* mutation) and MG1655 ptet-RFP *ompB-cat* (containing the WT *envZ*) mixed at initial ratios of 1:1, 1:100, and 1:1,000. The error bars represent the standard error of the mean of four mice.

We have shown that adaptive mutations play a critical role in the success of the E. coli MG1655 strain in colonizing of the mouse gut [12]. A possible clue to the nature of the mutation(s) selected during colonization ensued from our subsequent observation of bacteria with a reduced motility phenotype in the feces of all gnotobiotic mice colonized with the wild type MG1655 strain (WT) (Figure 1A). The colonies displayed a new small and granular morphotype (SG) distinct from the large and smooth morphotype (LS) of the WT inoculated strain (Figure S1). SG colonies forming bacteria, undetected in the initial inocula, appeared in the feces within two days, and reached a prevalence of 90% within seven days (Figure 1B). Their phenotype remained stable when grown in vitro over many generations, indicating that it was heritable and may result from the rapid in vivo selection of mutation(s).

In order to identify the potential mutations responsible for the SG morphotype, a clone forming SG colonies (SG1) isolated from mouse feces two days post-colonization, was transformed with a genomic DNA plasmid library generated from the parental WT strain. All plasmids that restored the ancestral WT LS morphotype carried the *ompB* locus, coding for the membrane sensor EnvZ and the transcriptional regulator OmpR of a two-component signal transduction system central to the osmolarity-dependent regulation of genetic expression [13]. A chloramphenicol resistance gene (*cat*), inserted downstream the *ompB* locus in the chromosome of the WT ancestral and the SG1 strains, co-transduced with a 95% frequency with the morphotype (LS or SG), indicating that in the SG1 strain, the DNA region surrounding *cat* was responsible for the SG morphotype. This region was sequenced for one SG and one LS clone harvested from the feces of each of the 8 independent mice inoculated with either MG1655 or an MG1655 E. coli strain carrying a yellow fluorescent protein (YFP) as reporter of *fliC* expression (MG1655*pfliC*-YFP) (see below). While no mutation was detected in LS clones, all SG clones displayed a different missense point mutation, seven located *in envZ*, and one in *ompR* (Table1). The independent systematic and rapid selection of mutations in the same genes under identical experimental conditions is evidence for a strong selective advantage of the mutants during gut colonization [1].

To confirm and estimate the relative fitness of the SG1 mutant versus the ancestral strain in the mouse gut, we performed in vivo competition experiments between strains isogenic except for the point mutation present in the *envZ* gene of the SG1 strain (SG1 mutation) and the inducible fluorescent marker (RFP vs. GFP). Prior experiments have indicated that these inducible markers do not induce any selection bias [14]. The ratio of mutant (GFP) to WT (RFP) colonies was defined after culture of the feces and ex vivo induction of the fluorescent marker. Competition experiments using initial ratios of mutant to WT strain of 1:1, 1:100 and 1:1,000 indicated that the SG1 mutation confers a considerable fitness gain (Figure 1C). With the assumption that the mean generation time for E. coli in the gut is 60 minutes [15], the selective advantage of the SG1 mutation was estimated to be 24% when the mutant to WT strain ratio remained under 1:10 (Table S1). These data explained how adaptive mutations in *envZ,* that are likely to happen at a frequency below 10^−7^, can be very rapidly selected upon colonisation with the WT strain. The selective advantage of the SG1 mutation decreased to approximately 10% when the ratio of mutant to WT strain increased over 1:10, indicating that the selective advantage conferred by the mutation is frequence-dependent, consistent with the observation that the WT strain is not entirely displaced in the mono-colonization experiment (Figure 1B).

### Selected EnvZ-OmpR Mutations Exert Pleiotropic Effects on Bacterial Motility and Permeability

Importantly, the selected mutants did not exhibit the same motility phenotype as null mutations, since strains deleted for *envZ*, *ompR* or both kept the wild type LS morphotype (Figure S1). The membrane receptor kinase-phosphatase EnvZ forms a two-component pair with its cognate response regulator, OmpR, that enable cells to sense external changes of osmolarity [13]. The native receptor exists in two active but opposed signalling states, the OmpR kinase-dominant state and the OmpR-P phosphatase-dominant state. The balance between the two states determines the level of intracellular OmpR-P, which in turn determines the level of transcription of the many target genes [13].

**Figure 2:**
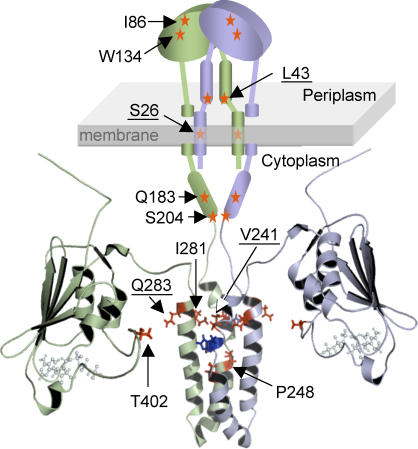
Localization of the Selected Mutations Mutations are depicted on a representation of EnvZ dimer, based on the transmembrane/periplasmic domain model [47] and the NMR structures of the homodimeric core [48] and the ATP-binding domain [49] with ATP bound molecules depicted in balls and sticks. Localizations of the amino acid where mutations had been identified are represented by red stars (linker and periplasmic domain) and with red side chain in the known EnvZ sub-structures. The side chain of active site His residues are in blue. Underlined amino acids are mutations previously described to increase the EnvZ kinase/phosphatase activity ratio in references [17] or [18].

One important bacterial function controlled by OmpR is motility, as OmpR regulates transcription of the *flhDC* operon, the master regulator of flagellar biosynthesis [16]. Several mutations identical to those selected in vivo during colonization were previously shown, by in vitro mutational analysis of EnvZ activities, to switch on the EnvZ kinase-dominant state [17,18] (Figure 2), resulting in increased levels of phospho-OmpR and repression of the *flhDC* operon [16].

**Figure 3:**
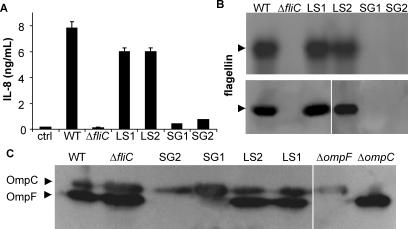
SG Mutants Lack Flagellin and Inflammatory Properties and Exhibit an Altered Porin Profile (A) IL-8 secretion measured by ELISA in supernatants of HT29-19A cells after a 16-h stimulation with WT, Δ*fliC*, SG (SG1 and 2), LS (LS1 and 2) strains, or medium alone (ctrl) (mean ± SEM for triplicates in one representative experiment out of five). (NF-κB DNA-binding activity and CCL-20 mRNA expression are presented in Figure S3). (B) Immunoblotting with an antibody against *E. coli* flagellin of proteins concentrated from culture supernatants (upper panel) or extracted from cell lysates (lower panel) of the same strains. (C) Immunoblotting with an antibody against *E. coli* OmpF and OmpC porins of outer membrane fraction of the same strains and control strains deleted of *ompF* or *ompC*.

Consistent with repression of flagellin expression in all SG mutants, no flagellin could be detected in cell lysates or supernatants obtained from stationary phase cultures, while the ancestral WT strain and the LS colonies (that kept the wild-type motility phenotype after mouse colonization) synthesised large amounts of flagellin in the same in vitro conditions (Figure 3B). We have previously shown that the WT ancestral E. coli strain induces a potent NF-κB-dependent inflammatory response in intestinal epithelial cells that hinges on the interaction of flagellin with Toll receptor 5 [19]. Consistent with impaired flagellin expression, culture supernatants of SG strains in stationary conditions, failed to induce any inflammatory signal in monolayers of epithelial cells (Figures 3A and S2).

**Figure 4:**
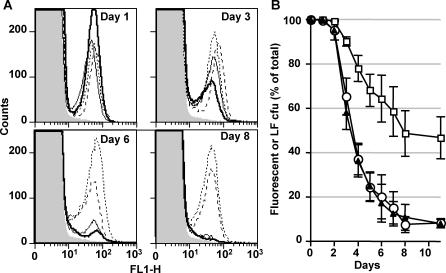
Flagellin Expression Is Counterselected during Colonization (A) *fliC* promoter activity monitored by flow cytometry: overlay fluorescence histograms in feces from germ-free mice (grey shading) and from two representative mice (see Figure S3 for the complete dataset) colonized with WT (solid lines) or Δ*fliC* (dotted lines) E. coli strains containing an YFP under the control of *fliC* promoter (p*fliC*-YFP), at different days post-inoculation. (B) Evolution over time (in days) of fluorescent cfu (circles) and LS cfu (filled triangle) in the feces of 12 mice inoculated with the WT p*fliC*-YFP strain (circles) and fluorescent cfu in the feces of 11 mice inoculated with the *ΔfliC* p*fliC*-YFP strain (square) (mean ± standard deviation).

These in vitro observations showing repression of flagellin synthesis in SG mutants were thus compatible with the observed defective motility morphotype. This morphotype was however clearly distinct from the pin point morphotype of the Δ*fliC* strain lacking the gene encoding flagellin, the primary flagellar subunit (Figure S1). In order to confirm that flagellin was downregulated by SG mutants in vivo in the intestine, germ-free mice were inoculated with an MG1655 E. coli strain carrying a yellow fluorescent protein (YFP) as reporter of *fliC* expression. The bacterial fluorescence in the feces was monitored in the feces by flow cytometry. Fluorescence decreased rapidly in mice inoculated with the WT strain, demonstrating in vivo down modulation of flagellin (Figures 4 and S3). Fluorescence monitoring after plating confirmed this result. Thus, in mice inoculated with the WT strain, the fraction of fluorescent colonies decreased to an average of 10% within 8 days, consistent with the selection of SG mutants described above (Figure 4B). Furthermore, all bacteria forming non-fluorescent colonies tested on motility plate exhibited an SG morphotype, while those forming fluorescent colonies retained the LS morphotype (Figure 4B).

As OmpR/EnvZ controls many activities, we looked for other effects of the selected mutants. The characteristic motility phenotype of the SG selected mutants could be a result of an enhanced aggregation of bacteria to each other via the production of curli fibres encoded by the *csgBA* operon whose expression is regulated by the OmpR regulated *csgD* gene [20]. However, in contrast to the previously described *ompR* mutant of E. coli K12 that promotes biofilm formation via the derepression of the *csgA* gene [21], none of the SG mutants exhibited changes in *csgA* gene expression and their biofilm formation was reduced compared to the WT strain (data not shown).

Another essential function of the two-component system envZ/ompR is to modulate membrane transport and permeability in response to medium osmolarity [22]. In particular, OmpR affects the reciprocal transcription of the small pore OmpC and large pore OmpF porins [23], the two E. coli porins that are thought to play a central role in the adaptation of *E.coli* to the hyperosmotic conditions of the intestine [24]. Consistent with mutations that switch on the OmpR kinase-dominant state of EnvZ, selected SG mutants had decreased *ompF* and increased *ompC* mRNA and membrane protein levels compared to the WT ancestral strain (Table 1, Figure 3C), *i.e.* a reduced permeability phenotype [23]. Membrane permeability is central for both stress protection and nutritional competence [25]. It has been postulated that reduced permeability would be favourable in the environmental conditions of the gut, consisting of high osmolarity, low oxygen pressure and the presence of bile salts [24]. Indeed, all SG mutants grew much better than the ancestor in medium containing bile salts, the ancestor being entirely displaced within 7 hours of growth (data not shown).

**Table 1:**
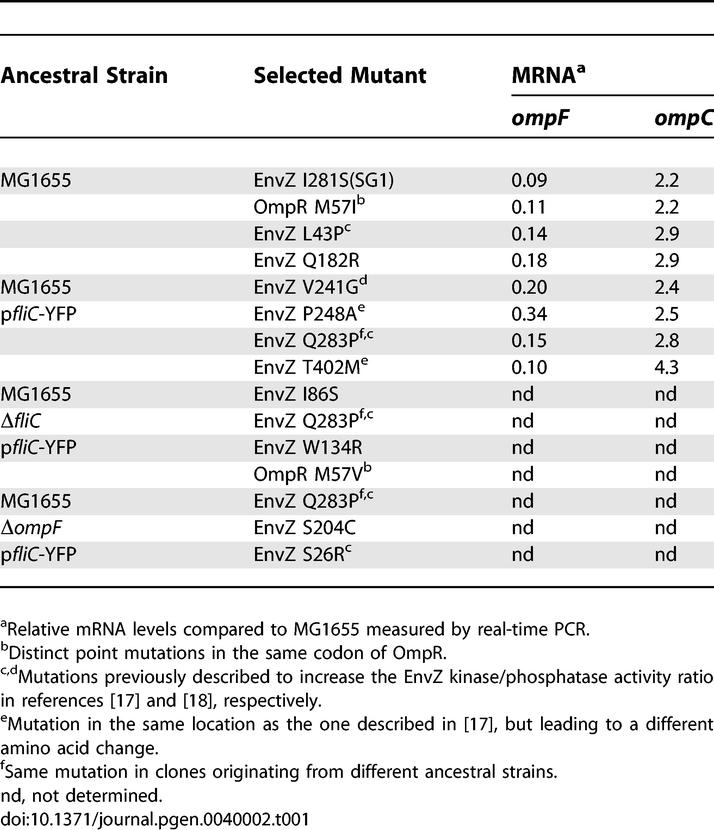
Characterization of Isolated Mutations

### Parallel Selection of EnvZ-OmpR Mutations Results from Additive Fitness Gains Conferred by Repression of Both Flagellin and OmpF Expression

Transcriptome analysis has pointed to the potential role of the two-component EnvZ/OmpR system in the regulation of multiple genes, including genes involved in transport across membranes and cell metabolism [22], which may perhaps promote intestinal adaptation of *E. coli.* We therefore assessed the importance of flagellin repression and/or porins regulation on the parallel selection of *envZ-ompR* mutations.

To analyse the role of flagellin in the selection of SG mutants, germ-free mice were inoculated with either the WT or the Δ*fliC* strain carrying a fluorescent protein (YFP) as reporter of *fliC* expression. Flow cytometry analysis of the feces showed that *in situ* fluorescence decreased faster and more extensively in mice inoculated with the WT than with the Δ*fliC* strain (Figures 4A and S3), a result confirmed by fluorescence monitoring after plating (Figure 4B). Thus, in mice inoculated with the Δ*fliC* strain, the fraction of fluorescent colonies had decreased to only 50% on day 8 as compared to 10% in mice inoculated with the WT strain and the kinetics of selection was slower (Figure 4B). Altogether, these results point to a strong impact of flagellin on the selection of EnvZ mutations. However, mutations downregulating *fliC* expression could still be selected despite the absence of flagellin, presumably because of the pleiotropic effect of these mutations. Sequencing the *ompB* locus in non-fluorescent clones harvested from 4 mice inoculated with the Δ*fliC* strain revealed missense point mutations (Table 1). Three were located in *envZ,* including one identical to a mutation found in a clone isolated from a mouse inoculated with the WT strain. The fourth one was located in the same codon of *ompR* as the mutation identified in a clone derived from the WT strain (Table 1). These results show that the adaptive advantage conveyed by selected mutations is only partially flagellin-dependent, suggesting that selected mutations provide further advantage resulting from the modulation of other genes controlled by OmpR.

**Figure 5:**
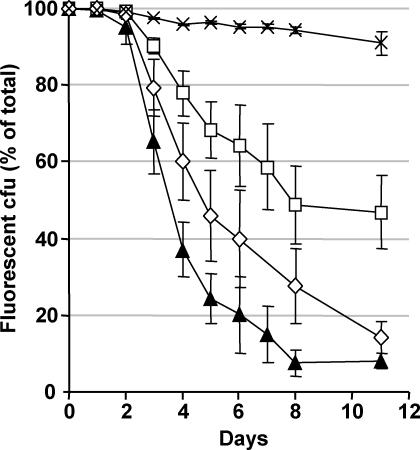
In Vivo Selection of Mutations during Intestinal Colonization Results from Two Independent and Additive Effects on FliC and OmpF Expression Evolution over time (in days) of non-fluorescent cfu in the feces of mice inoculated with the WT p*fliC*-YFP strain (filled triangles, *n* = 12), the Δ*fliC* p*fliC*-YFP strain (empty squares, *n* = 11), the Δ*OmpF* p*fliC*-YFP strain (empty diamonds, *n* = 5), or the double mutant Δ*ompF* Δ*fliC* p*fliC*-YFP strain (crosses, *n* = 4) (mean ± standard deviation).

**Figure 6:**
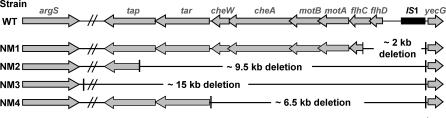
Deletions in the Region Downstream of *flhDC* Operon Are Selected for in Mice Colonized with the Δ*OmpF*p*fliC*-YFP Strain Genetic map of the deletions detected in four non-motile clones isolated from four independent mice. All non-motile clones tested possess a deletion in the *flhDC* region.

One likely candidate was the large porin encoding gene *ompF*. Indeed we have observed that this gene expression is downmodulated by the selected *envZ-ompR* mutations, resulting in a reduced permeability phenotype known to be associated with increased resistance to bile salts [26], as observed for SG mutants. To assess the role of OmpF in the selection of EnvZ mutations, mice were inoculated with a Δ*ompF* mutant that expresses OmpC but no OmpF protein (Figure 3C) and carries the YFP reporter of *fliC* expression. Although the impact of OmpF deletion alone was not as strong as the one of flagellin, selection of non fluorescent mutants studied in the feces after plating was significantly less efficient than in mice colonized with the WT E. coli strain (Figure 5). In one out of five studied mice, all non-fluorescent mutants exhibited an SG phenotype in soft agar plates. In two other mice, the non-fluorescent colonies had a totally nonmotile (NM) pinpoint phenotype comparable to the Δ*fliC*-engineered strain (Figure S1). In the last two mice, both SG and NM morphotypes were observed. Sequencing the *ompB* locus revealed a missense mutation in *envZ* in all SG clones tested (Table 1). In contrast, NM clones forming pin-point colonies had a normal *envZ* sequence but contain large deletions from 1.5 to 12 kb between the *otsA* and *cheB* loci, encompassing the *flhDC* operon and thereby precluding any expression of the whole flagellum operons (Figure 6). Interestingly, all deletions had occurred immediately upstream of an Insertion Sequence (IS1) located just upstream the *flhDC* operon, and probably reflecting an imprecise excision of the IS [27]. The deleted genes, that all belong to the chemotaxis/motility pathway, failed to be amplified by PCR (data not shown), showing that they were indeed lost rather than inserted ectopically**.** These results show that in mutants with reduced permeability, the major fitness gain results from repression of gene(s) controlled by FlhDC, probably flagellar genes and in particular the *fliC* gene encoding flagellin.

To confirm this hypothesis, mice were inoculated with double Δ*fliC* Δ*ompF* mutants carrying the YFP reporter of *fliC* expression and expressing the fluorescent CFP protein under the control of a constitutive promoter. Strikingly, combining deletions of porins and flagellin had additive effects and almost entirely abolished the in vivo selection of EnvZ/OmpR mutants (Figure 5). At day 11 post-inoculum, only 9% of clones were YFP-negative. None had mutation in the *ompB* locus, a deletion in the *flhDC* region or a mutation in the p*fliC*-YFP construct. These YFP-negative clones were all CFP positive and remained CFP positive during the 100 days of observation. Since YFP and CFP were expressed at the same level in the inoculated strain, the hypothesis that YFP expression was costly for the bacteria and eliminated by mutations is unlikely.

Therefore, our results show that the selective pleiotropic advantage conferred EnvZ/OmpR mutation predominantly results from a combined effect of modulation of *fliC* expression and membrane permeability, but does not exclude minor additional effect(s) of (an)other as yet uncharacterized gene(s) under the control of EnvZ/OmpR.

## Discussion

Due to their high growth rate and large population size, microbes have a remarkable capacity to evolve and diversify by generation and spread of mutations that improve their fitness in a given environment [1]. We have previously observed that within a few days a mutant strain with a high mutation rate increased in frequency to the expense of the parental commensal E. coli MG1655 strain during gut colonization. In contrast, the mutator strain lost the competition against a clone collected from the feces of mice colonized for 40 days with the parental commensal E. coli MG1655 [12]. These results suggested that adaptive mutations enable bacteria to rapidly and efficiently cope with the drastic environmental changes encountered during gut colonization. Our novel results identify the central role of the EnvZ/OmpR regulon in the physiological adaptation of *E. coli MG1655* to the gut environment, and show that adaptive mutations in this two-component system provide an additional gear to adjust precisely the scale of the physiological regulation controlled by this regulator to the gut environment. Furthermore our results provide the molecular basis of the beneficial effects of the pleiotropic mutations in EnvZ/OmpR in adaptation of E. coli MG1655 to the mouse gut.

Mutations in the *envZ/ompR* locus were systematically detected in 90% of bacteria harvested from independent mice feces within a week of colonization with WT E. coli MG1655. Except for one mutation in its cognate transcription factor OmpR, all mutations were found in the membrane sensor EnvZ. The major fitness gain conferred by these mutations was confirmed by in vivo competitions between the ancestor WT strain and an isogenic mutant strain harboring the prototype SG1 e*nvZ* mutation. The emergence of distinct point mutations at the same two-component locus in bacterial populations evolving in different colonized mice suggested a comparable impact on the physiological effects mediating the fitness gain due to these mutations. Indeed all mutations resulted in profound repression of flagellin expression and modulation of OmpF versus OmpC porin expression yielding a reduced permeability phenotype. This phenotype is typical of mutations that switch the phosphatase/kinase membrane sensor EnvZ toward a OmpR kinase-dominant state. Indeed several of the missense mutations selected during in vivo colonisation were previously identified by in vitro mutational analysis as turning on this functional state [17,18]. Mutations selected during colonization were not restricted to the catalytic domains of EnvZ, but were also found in the periplasmic sensor and cytoplasmic linker domains, highlighting the participation of all of the protein's domains in the control of gene regulation (Figure 2). Interestingly in mice colonized with the Δ*fliC* mutant, where adaptive mutants were mainly selected on their reduced permeability phenotype, mutations were still exclusively found in the EnvZ/OmpR system, a result that underscores the prominent role of the EnvZ/ompR system in the regulation of membrane permeability of E. coli MG1655 during intestinal colonization.

Notably, colonization with the WT E. coli did not select for mutations inactivating genes specifically controlling motility or permeability. Yet, selection of mutants with deletion of *flhD/C* operon was observed during colonization by the Δ*ompF* strain, a result reminiscent of observations in streptomycin-treated mice [28,29]. Clonal interference [30] thus likely prevents the selection of mutations affecting only one function, presumably associated with smaller selective value than the pleiotropic mutations in *envZ/ompR* modulating simultaneously functions as different as permeability and motility. Indeed, using reporter mutant bacteria carrying a fluorescent protein under the control of the *fliC* promoter, we could clearly demonstrate that the selective advantage conveyed by mutations in *envZ/ompR* resulted from their pleiotropic and additive effects on the repression of flagellin production and OmpF porin expression. The almost complete abolition of adaptive selection of *envZ/ompR* mutations in mice colonized with a double mutant E. coli strain that lacks both *fliC* and *ompF*, underscores the major contribution of the pathways controlled by *envZ* and *ompR* in the intestinal adaptation of E. coli. The precise elucidation of the selective forces is beyond the scope of this study, but likely scenarios are briefly discussed below. Flagellin downregulation could be selected for via its pro-inflammatory role [19,31–35], via its direct energetic cost [28,36], or via still non-identified mechanisms. The fitness gain conveyed by reduced permeability was suggested by in vitro analysis indicating that, similar to Δ*ompF* mutants, all *ompR/envZ* mutants grew much better in medium containing high concentrations of bile salts, a major stress factor for bacteria in the intestinal lumen. Interestingly, it has been reported that the concentration of biliary salts in the intestinal lumen decreases upon colonization [37,38]. A lower concentration of biliary salts in mice treated by streptomycin which empties the *enterobacteriae* niche but does not deplete completely the intestinal flora, might explain the predominant selection of mutants in the *flhD/C* operon in this mouse model [28,29].

In E. coli, stress protection comes at the cost of nutritional competence through the regulation of membrane permeability [39]. In the gut rich environment, bacterial nutrient intake is likely sufficient even if permeability is restrained, so that the growth rate is not significantly affected [25]. Yet, the extent of physiological regulation allowed by wild type EnvZ/OmpR might not be optimal to respond to our experimental mice gut conditions. Thanks to adaptive mutations in EnvZ/OmpR, the trade-off between self-preservation and nutritional competence (SPANC balance) might easily be switched to either better resistance or faster growth [25]. To mutate may thus represent a complementary genetic gear to adjust precisely the scale of physiological regulation controlled by a global regulator when switching between complex environments.

Notably, the selective advantage conferred by the *envZ m*utations was frequency dependent, consistent with the observation that in mice colonized with the WT strain, the mutation invades rapidly and massively the population, but does not go to fixation, as a minor part of the population kept the original colony morphotype (and genotype for envZ-ompR). These results suggest a mechanism causing the coexistence of ancestral and evolved form, perhaps because the ancestral phenotype confers some advantage to colonize a specific niche. Work is in progress to address this issue.

Experiments with microbial populations have been largely used to gain insight into the mechanics of evolution and have pointed to the possible important role of pleiotropic adaptive mutations [1]. Thus, finding mutations in regulatory genes is a recurrent observation both in natural populations and during in vitro experimental evolution, that led to postulate that mutations affecting regulators are more likely to promote adaptation and evolution than those improving a single enzymatic step [1,25,40]. Our results obtained in an in vivo model of bacterial evolution supports this hypothesis. As mutations in global regulators affect the regulation of many genes, they must be pleiotropic and are thus expected to result in the expression not only of beneficial but also of detrimental traits. The molecular mechanisms responsible for the selection of such pleiotropic mutations have therefore remained largely elusive in most systems. A recent study in a simple ecological in vitro model [41], has shown that adaptive mutations allow P. fluorescens to occupy a novel ecological niche at the air-liquid interface [42]. All selected strains had pleiotropic loss-of-functions mutations in one gene encoding a putative methyl-esterase in the *wsp* operon [2,3]. Drawing analogy with the *che* operon of E. coli that encodes proteins homologous to the *wsp* operon, the authors suggested that this protein acts in concert with a putative methyl-transferase to adjust the activity of a kinase. The mutations may thus destroy the capacity of the pathway to fluctuate between activity states, producing instead a steady state output allowing niche specialization. Our results, combined with previous biochemical works [17], provide direct evidence that a distinct scenario promotes the in vivo adaptation of an E. coli MG1655 to the gut of germ-free mice. In the case of EnvZ/ompR, the two opposed enzymatic activities are exerted by the cytoplasmic domain of EnvZ and are modulated in response to signals sensed by the external domain of the protein. Mutations in EnvZ, that directly affect the balance between two activities, are selected because of their independent and additive effects on genes controlling flagellin expression and membrane permeability. Dissecting the fitness gain due to these independent pathways allowed us to demonstrate that the EnvZ/OmpR global regulator orchestrates the physiological adaptation of *E. coli MG1655* to the gut environment. More generally, the observation that the EnvZ/OmpR system gathers under its control genes central to promote intestinal colonization leads us to suggest that global regulators may have arisen during evolution to optimize the coordination of genes that collaborate to adapt to a given niche. Mutations in such global regulators may provide a complementary genetic tool that allows bacteria to extend the scale of the physiological regulation and promotes their rapid adaptation when confronted to very specific environments.

## Materials and Methods

### Bacterial strains.

All strains were derived from the commensal flagellated *E. coli* K12 MG1655 sequenced strain [43]. The MG1655 Δ*fliC E. coli* isogenic mutant has been described [19]. To construct the reporter WT p*fliC*-YFP strain used to monitor in vivo activity of *fliC* promoter, sequence encoding the fluorescent protein YFP++ [44] was cloned downstream the upstream region of the *fliC* gene (p*fliC*: from nucleotides −230 to +5 relative to the translation start). The fragment (p*fliC* YFP, T1T2 and *cat*) was flanked by 40 nucleotides sequences homologous respectively to the 5′ and 3′ of the IS2 and IS30 insertion sequences interrupting the *ybdA E. coli* gene and by KpnI and SphI restriction sites and cloned in p5Y, a pUC-18-derived plasmid. After plasmid amplification, the fragment was inserted into the *ybdA* gene of the MG1655 E. coli chromosome replacing the IS sequences following method already described [45]. MG1655 Δ*fliC* p*fliC*-YFP was constructed by P1 phage co-transduction of the p*fliC*-*YFP*-v^+^ and the *cat* alleles from MG1655 p*fliC*-YFP into MG1655 Δ*fliC* strain.

The MG1655 *ompB-cat* and SG1 *ompB-cat E. coli* strains (used to assess the link between the *ompB* locus and the motility phenotype) were constructed by inserting the FRT flanked *cat* gene of the pKD3 plasmid [45] between the *envZ* and *pck* genes as described [45], using PCR primers that contained a 40 bases-5′ end extension centered on the translation stops of the *envZ* or *pck* gene. Insertion of the PCR product was monitored using primers respectively identical or complementary to the nucleotides 1562 to 1582 of *pck* and 1238 to 1258 of the *EnvZ* gene. These strains kept the motility phenotype of the MG1655 and SG1 strains respectively.

The MG1655 ptet-GFP *ompBSG1-cat* and MG1655 ptet-RFP *ompB-cat E. coli* strains (used to measure the relative fitness of the SG1 strain in vivo) were constructed by introducing by P1 phage co-transduction of the *ompB* region from the SG1 *ompB-cat* strain and the *cat* allele into the MG1655 ptet-GFP and the MG1655 ptet-RFP strains respectively (described in [14]). The MG1655 ptet-GFP *ompBSG1-cat* strain was selected among granulous transductants (SG morphotype) in motility agar whereas the MG1655 ptet-RFP *ompB-cat* was selected among transductants that kept the WT motility phenotype (LS morphotype).

The Δ*ompF*, Δ*ompC*, Δ*ompR*, Δ*envZ*, and Δ*ompB* strains were constructed by replacing the *ompF*, *ompC, ompR*, *envZ* and *envZ* and *ompR* open reading frame respectively from start to stop codon by the FRT flanked *cat* gene of the pKD3 in the E. coli MG1655 strain following method already described [45]. The MG1655 Δ*ompF* p*fliC*-YFP strain was constructed by P1 phage co-transduction of the Δ*ompF* and the *cat* alleles from MG1655 Δ*ompF* into MG1655 p*fliC*-YFP p2*rrnB*-CFP strain. The MG1655 Δ*fliC* Δ*ompF* p*fliC*-YFP strain was constructed by P1 phage co-transduction of the Δ*ompF* and the *cat* alleles from MG1655 Δ*ompF* into MG1655 Δ*fliC* p*fliC*-YFP p2*rrnB*-CFP strain.

To construct the reporter p2*rrnB*-CFP, sequence encoding the fluorescent protein CFP++ was cloned upstream of the promoter p2 of the *rrnB* operon (p2*rrnB*: from nucleotides 152 to 94 relative to the translation start of the *rrsB* gene). The fragment (p*rrnB*-*cfp*, T1T2 and *cat*) was flanked by 40 nucleotides sequences homologous respectively to the 5′ and 3′ of the *IntC* (*IntS*) E. coli gene and by KpnI and PacI restriction sites and cloned in a pUC-18-derived plasmid. After plasmid amplification, the fragment was inserted into the *IntC* gene of the MG1655 E. coli chromosome following method already described [45].

### Genomic library.

Genomic DNA from E. coli MG1655 strain was prepared with the Wizard Genomic DNA Preparation kit (Promega, Charbonnières, France) and partially digested with the Sau3AI restriction enzyme. Fragments ranging from 2 to 6 kb were eluted from agarose gel (Gel extraction kit, Promega), and cloned into BamHI-digested and dephosphorylated pACYC184 plasmid. The purified ligation reaction was used to electro-transform DH5-α E. coli. Transformants were selected on LB plates containing chloramphenicol. Ligation efficiency was 95% and average size of genomic inserts 3 Kb. Plasmids were extracted from about 1.5 × 10^4^ pooled colonies (Miniprep kit, Promega). The SG1 clone was transformed with the genomic library and transformants were selected on motility plates supplemented with chloramphenicol. The clones with a wild type motility phenotype (LS) were isolated and the *E.coli* MG1655*-*derived locus carried by the transforming plasmids was determined by sequencing with primers flanking the cloning site.

### Sequence.

Sequencing of the *ompB* locus (from the *greB* translation stop to the *pck* translation stop) and p*fliC*-YFP construction of the MG1655 strain and of the clones isolated from mouse feces was carried out on purified PCR amplification products using standard procedures in the Institut Cochin sequence facilities.

### Determination of the sizes of the deletions.

The following primers were used to define the size of the deletions in MG1655 Δ*ompF* p*fliC*-YFP non motile (NM) mutants: otsAup (5′-GTGCAACTCAGGCATCATGG-3′) either in association with CheBdwn (5′-CGTATGGTGGAAAAGTCATCC-3′) for clones NM2 and NM4, with CheAdwn (5′-cgctgaagccaaaagttcctgc-3′) for the clone NM1 and with ArgSdwn (5′-CTAACGGCATGATGGGAGTTG-3′) for clone NM3.

### Bacterial counts and motility.

Bacterial motility was monitored in soft agar plates (4.5 g/L agar in Luria broth medium (LB)) at 30 °C for 24 h. Enumeration of fluorescent bacteria was made on solid LB agar plates (15 g/L) after a 48-h incubation at 37 °C using a lighting system (LT-9500–220 Illumatool, Lightools Research). YFP fluorescence was detected in colonies using 470-nm excitation wavelengths and 530-nm reading filters. Fluorescence detection in feces was performed on dilutions of freshly passed feces using a BD-LSR flow cytometer (Becton Dickinson). Data were analyzed with Cell Quest software (Becton Dickinson).

### Bacterial competition in the presence of bile salts.

Strains were grown in LB for 16 h, and population sizes were determined by plating appropriate dilutions of the culture on LB plates. 50 μL of a 1 × 10^4^ fold dilution of the pre-culture of the mutant and of the reference parental strain were inoculated in 5 mL of LB and LB supplemented with bile salts (Bile salts N°3, Difco) at 5% (M/W) and incubated at 37 °C under agitation. Mutant and parental population sizes were determined after 7h30 of culture by counting SG and LS populations on motility plates (for SG1 and SG2 against MG1655 competitions), or fluorescent and non-fluorescent populations as described above.

### Mice and in vivo colonization experiments.

Conventional and germ-free C3H/HeN mice were bred at the INRA facilities. Germ-free and gnotobiotic mice were reared in isolators (Ingenia) in individual cages and fed *ad libitum* on a commercial diet sterilized by gamma irradiation (40 kGy) and supplied with autoclaved (20 min, 120 °C) tap water. For colonization experiments, 8–12-week-old germ-free mice were inoculated *per os* with 10^4^ bacteria from the chosen strain in 0.5 mL 10^−2^ M MgSO_4_. Colonization was monitored by bacterial counts in individual freshly harvested fecal samples as described [12].

For in vivo bacterial competition, MG1655 ptet-GFP *ompBSG1-cat* (containing the *envZ* SG1 I281S mutant allele) and MG1655 ptet-RFP *ompB-cat E. coli* (containing the *envZ* wild type allele) were grown in LB for 16 h and mixed at the 1:1, 1:100, 1:1,000 SG to WT ratios. 0.5 mL of a 1 × 10^4^ fold dilution in 10^−2^M MgSO_4_ of these mix were used for mouse colonization. Mutant and WT population sizes were determined every 12 h by counting Red and Green fluorescent CFU on plates containing anhydrotetracycline (50 μM Acros) during 5 days following colonization. The maximal relative fitness was estimated by fitting an exponential curve to the evolution of the SG/WT ratio between 12 and 36 hours following colonization with the initial ratios 1:100 and 1:1,000.

All procedures were carried out in accordance with the European guidelines for the care and use of laboratory animals.

### Cell culture and bacterial stimulation.

Stimulation of monolayers of the human IEC line HT29-19A with live bacteria, preparation of epithelial cell nuclear extracts, electrophoretic mobility shift assay (EMSA), determination of CCL-20 mRNA level by real-time quantitative PCR after a 6-h stimulation and determination of IL-8 concentrations in epithelial cell supernatants by enzyme-linked immunosorbent assay (ELISA) (Duoset kits, R&D Systems) were all performed as previously described [19].

### cDNA synthesis and real-time PCR.

Total RNA was extracted from 5 ml of stationnary phase culture (at 37 °C, with agitation) using the RNeasy kit (Qiagen), according to the manufacturer's instructions. RNA was treated with four units of the Turbo DNA-free (Ambion) for 1h at 37 °C. RNA was quantified by measuring the optical density at 260 nm and checked for degradation by an agarose gel electrophoresis. The cDNA synthesis was performed using 2 μg RNA with random hexamers (12.5 ng/ml) and the Superscript II RNAse H^−^ kit 5 (invitrogen) according to the manufacturer's instructions.

The real-time PCR experiments were performed using the SYBRgreen PCR Master Mix (Applied Biosystems) to quantify the expression level of the *ompC* and *ompF* genes. The r*poD* gene was chosen as a reference gene for data normalization. The primers RpoD1RT (5′-GTAGTCGGTGTTCATATCGA-3′), RpoD1FT (5′-CGTCTGATCATGAAGCTCT-3′), OmpC2RT (5′-GTCAGTGTTACGGTAGGT-3′), OmpC2FT (5′-CGACTACGGTCGTAACTA-3′), OmpF2RT (5′-CCTGTATGCAGTATCACCA-3′) and OmpF2FT (5′-CCAGGGTAACAACTCTGAA-3′) were designed by the Primer Express software (Applied Biosystems). Amplification and detection of the specific products were carried out with the 7300 Real Time PCR System (Applied Biosystems). Data analysis was performed with the 7300 System Software. For each target gene, the average Ct value was calculated from triplicate reactions for RNA samples. The difference between Ct of the target gene and Ct of the endogenous reference gene (*rpoD*) was defined as the ΔCt. The ΔΔCt value described the difference between the ΔCt of the wild type strain and the mutant strain. The difference in expression was calculated as 2^ΔΔCt,^ and a twofold difference was considered as significant.

### *FliC* western blotting.

Bacterial proteins were obtained from culture supernatants precipitated by 10% trichloroacetic acid and from bacterial pellets sonicated in PBS containing an anti-protease cocktail (Roche Diagnostics) and 1% Triton X100 (Sigma). Twenty μL of 25-fold concentrated bacterial supernatants or 20 μg of total proteins from bacterial lysates were electrophoresed on 10% SDS-PAGE gels and transferred onto PVDF membranes (Amersham Biosciences, Saclay, France). Membranes blocked with 5% nonfat dry milk in 20 mM Tris pH 7.5, 150 mM NaCl, and 0.05% Tween-20, were incubated overnight with a 1:2,000 dilution of monoclonal antibody 15D8 against E. coli flagellin (Bioveris Europe), and then for 1 h with a 1:8,000 dilution of HRP-conjugated goat anti-mouse immunoglobulins (Amersham Biosciences). HRP was revealed with ECL-Plus light (Amersham Biosciences) using a luminescent image analyzer LAS-1,000plus (Fujifilm).

### *OmpF* and *OmpC* western blotting.

Cultures (20 ml) grown at 37 °C with agitation were harverested and washed in 20 mM sodium phosphate buffer, pH 7.4. The pellet were suspended and sonicated in 10 mM Hepes buffer, pH 7.4 (Vibra-cell, Bioblock Scientific). Sarkosyl was added to a final concentration of 0.5% and the detergent extraction was carried out at room temperature for 1 hour. The unbroken cells were removed by centrifugation at 3,000 rpm for 10 min, and the outer membrane fraction was obtained by an ultracentrifugation at 40,000 rpm for 1 hour. The outer membrane proteins were suspended in 10 mM Hepes and quantified by Bradford Method (Biorad). Samples were analyzed by SDS-polyacrylamide gel electrophoresis containing 8M urea as described previously [46]. Gels were transferred onto PVDF membranes at 200 mAmp for 50 minutes. Membranes blocked with 5% nonfat dry milk in 20 mM Tris pH 7.5, 150 mM NaCl, and 0.05% Tween-20, were incubated overnight with a 1/1,000 dilution of an rabit anti-OmpC/F (gift from Roland Lloubès, CNRS UPR 9027, Institut de Biologie Structurale et Microbiologie, Marseille), and then for 1 h with horseradish peroxidase conjugated anti-rabbit immunoglobulins (Cell Signaling Technology).

## Supporting Information

Figure S1 Motility Morphotypes on Soft Agar Plates WT: large and smooth morphotype of the MG1655 strain. Δ*fliC*: pin point morphotype of an isogenic strain carrying a deletion in the *fliC* gene and therefore non-motile. Δ*ompR*: large and smooth morphotype comparable to that of the WT strain. SG1: small and granular morphotype of a clone isolated 2 d after inoculation from the feces of a mouse colonized with the WT strain; NM1: non-motile morphotype of a clone isolated 8 d after inoculation from the feces of a mouse colonized with the ΔompF pfliC-YFP strain. Soft agar plates after a 24-h incubation at 30 °C.
(1.2 MB PDF)

Figure S2 Clones Forming SG Colonies Fail to Induce an Inflammatory Response in an Intestinal Epithelial Cell Line (A) NF-kB DNA-binding activity assessed by EMSA as described [19] for HT29-19A cells stimulated 3 h with MG1655 (WT), Δ*fliC* strain (Δ*fliC*), and clones forming SG (SG1 and 2) or LS (LS1 and 2) colony or medium alone (ctrl). Data are representative of five experiments.
(B) CCL-20 mRNA expression measured by real time RT-PCR on RNA extracted from 6-h stimulated cells (mean ± SEM for triplicates in one representative experiment).
(66 KB PDF)

Figure S3 *fliC* Promoter Activity Monitored by Flow Cytometry Overlay histograms of fluorescence level in feces from germ-free mice (gray shading) and from mice colonized with WT pfliC-YFP or Δ*fliC* p*fliC*-YFP E. coli at different days post inoculum. (A) and (B) are the results of two experiments run at different times in the same conditions with six mice inoculated with the p*fliC*-YFP E. coli (WT) and six (A) or five (B) mice inoculated with the Δ*fliC* p*fliC*-YFP E. coli (Δ*fliC*).
(1.6 MB PDF)

Table S1 Selective Advantages Conferred by the envZ SG1 Mutation during Gut Colonization (48 KB DOC)
