# Supplementary material for: Dissecting the Genetic Components of Adaptation of Escherichia coli to the Mouse Gut
Source: PLoS Genet. 2008 Jan 11;4(1):e2. doi: 10.1371/journal.pgen.0040002 (PMC2174974; doi:10.1371/journal.pgen.0040002)

FIGURE S1

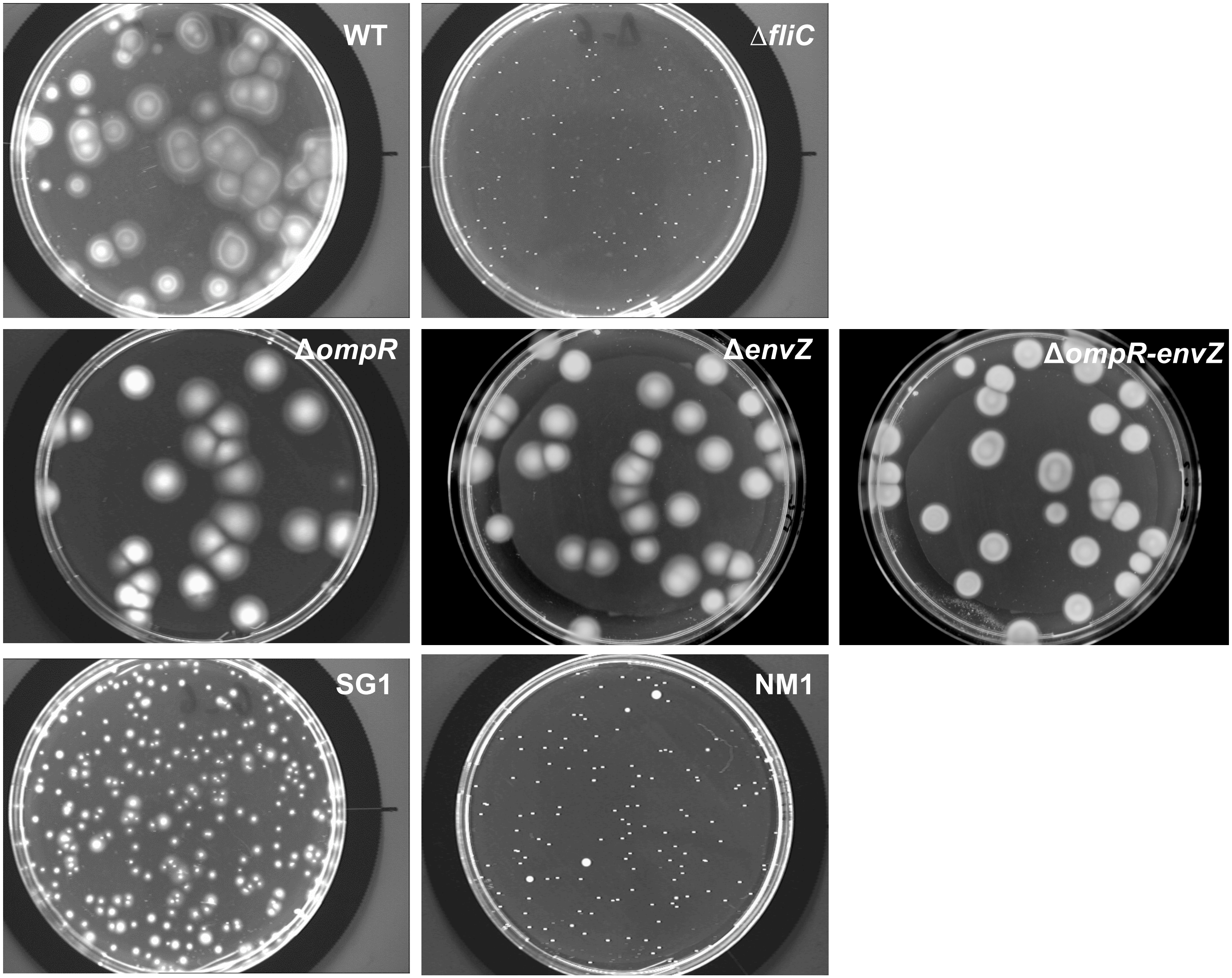

Supplement: Supplementary file 1 [file pgen.0040002.sg001.pdf]
