# Supplementary material for: Dissecting the Genetic Components of Adaptation of Escherichia coli to the Mouse Gut
Source: PLoS Genet. 2008 Jan 11;4(1):e2. doi: 10.1371/journal.pgen.0040002 (PMC2174974; doi:10.1371/journal.pgen.0040002)
Supplement: Supplementary file 2 [file pgen.0040002.sg002.pdf]

FIGURE S2

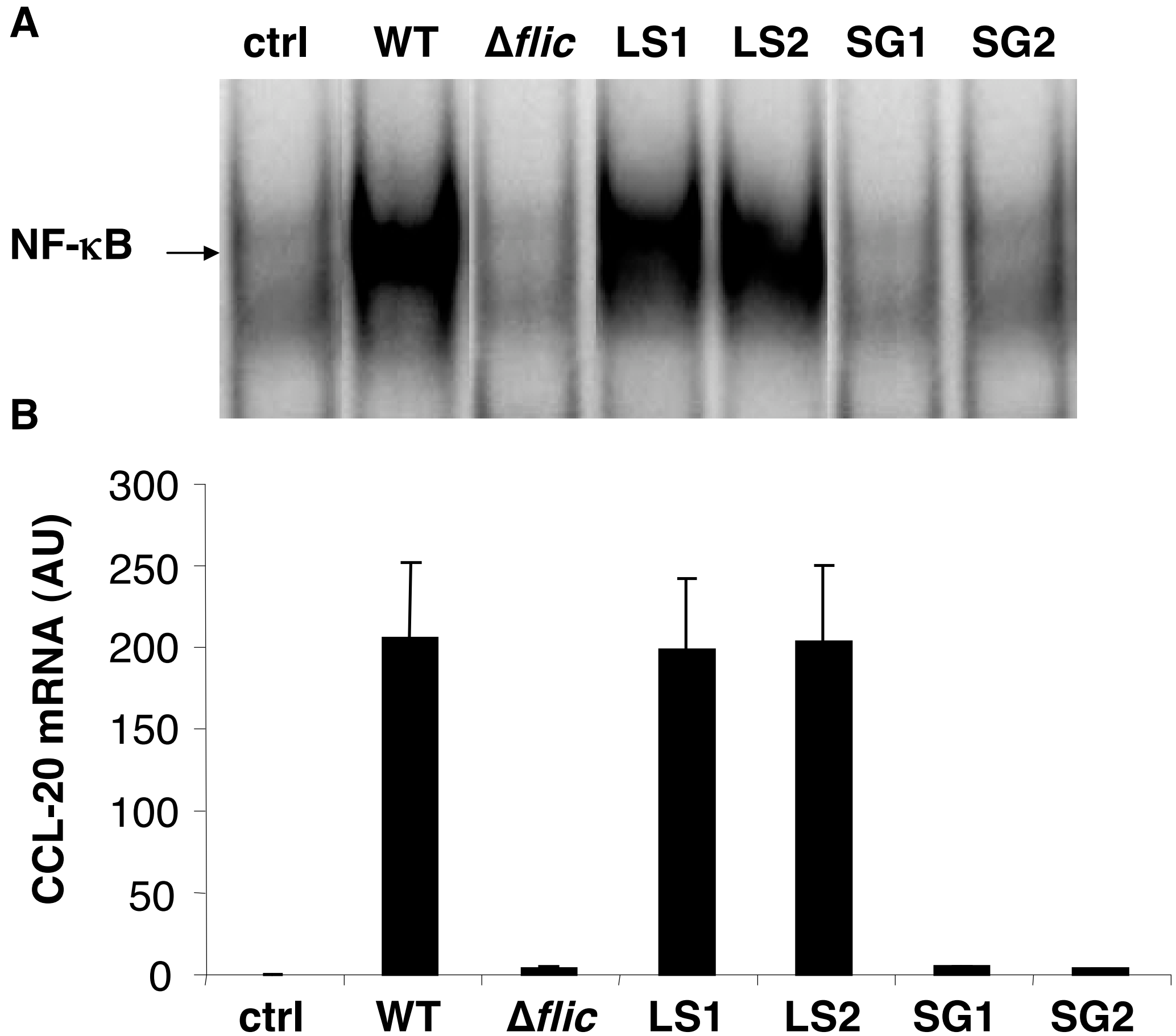
A
ctrl
WT
Δ*flic*
LS1
LS2
SG1
SG2
NF-κB
B
CCL-20 mRNA (AU)
300
250
200
150
100
50
0
ctrl
WT
Δ*flic*
LS1
LS2
SG1
SG2
