# Supplementary material for: Dissecting the Genetic Components of Adaptation of Escherichia coli to the Mouse Gut
Source: PLoS Genet. 2008 Jan 11;4(1):e2. doi: 10.1371/journal.pgen.0040002 (PMC2174974; doi:10.1371/journal.pgen.0040002)
Supplement: Supplementary file 3 [file pgen.0040002.sg003.pdf]

FIGURE S3

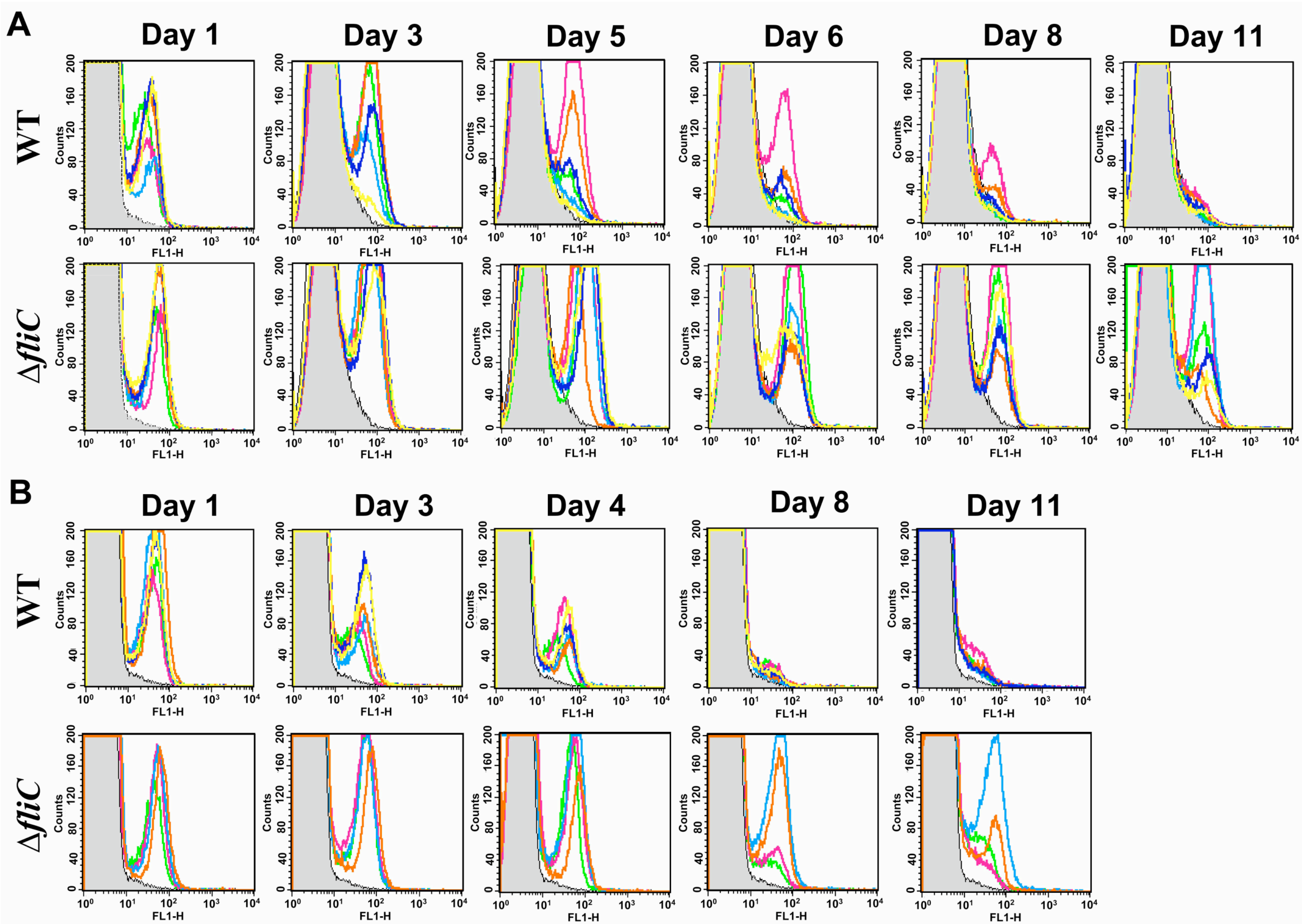
A
Day 1
Day 3
Day 5
Day 6
Day 8
Day 11
WT
ΔfliC
Counts
FL1-H
B
Day 1
Day 3
Day 4
Day 8
Day 11
WT
ΔfliC
Counts
FL1-H
