# Supplementary material for: Dissecting the Genetic Components of Adaptation of Escherichia coli to the Mouse Gut
Source: PLoS Genet. 2008 Jan 11;4(1):e2. doi: 10.1371/journal.pgen.0040002 (PMC2174974; doi:10.1371/journal.pgen.0040002)
Supplement: Supplementary file 4 [file pgen.0040002.st001.doc]

Supporting TableS1

| Initial SG1/WT ratio | SG1/WT <10 | SG1/WT >10 |
| --- | --- | --- |
| 1 |  | 10% [7-14] |
| 0.01 | 24% [16-33] | 11% [7-14] |
| 0.001 | 24% [16-32] | 11% [7-15] |

**Table S1: Selective advantages conferred by the envZ SG1 mutation during gut colonization**. The selective advantages have been estimated during the first days of colonization in two different ranges of the SG1 to WT strains ratios: when the SG1 mutants represent less than 90% of the population (ratio<10) and when they represent more than 90% of the population (ratio>10). The mean bacterial generation time used is 60 min, as evaluated in [15]. Between brackets are indicated the selective advantages obtained respectively with generation times of 40 and 80 minutes, which represent the extreme values estimated in [15]. The selective advantage is the slope of the regression of SG1/WT cfu expressed as a function of the number of generations.
